# Test-retest reliability of the ‘Reading the Mind in the Eyes’ test: a one-year follow-up study

**DOI:** 10.1186/2040-2392-4-33

**Published:** 2013-09-11

**Authors:** Enrique G Fernández-Abascal, Rosario Cabello, Pablo Fernández-Berrocal, Simon Baron-Cohen

**Affiliations:** 1 Department of Basic Psychology, Faculty of Psychology, The National Distance Education University (UNED), Madrid, Spain; 2 Department of Developmental Psychology and Education, Faculty of Education Science, University of Huelva, Huelva, Spain; 3 Department of Basic Psychology, Faculty of Psychology, University of Malaga, Campus Teatinos, s/n, 29071, Malaga, Spain; 4 Autism Research Centre, Department of Psychiatry, Cambridge University, and CLASS Clinic, Cambridgeshire and Peterborough NHS Foundation Trust (CPFT), Cambridge, UK

**Keywords:** Reading the mind in the eyes, Reliability, Assessment, Social cognition, Theory of mind

## Abstract

**Background:**

The ‘Reading the Mind in the Eyes’ (Eyes) test is an advanced test of theory of mind. It is widely used to assess individual differences in social cognition and emotion recognition across different groups and cultures. The present study examined distributions of responses and scores on a Spanish version of the test in a non-clinical Spanish adult population, and assessed test-retest reliability over a 1-year interval.

**Methods:**

A total of 358 undergraduates of both sexes, age 18 to 65 years, completed the Spanish version of the test twice over an interval of 1 year. The Bland-Altman method was used to calculate test-retest reliability.

**Results:**

Distributions of responses and scores were optimal. Test-retest reliability for total score on the Eyes test was .63 (*P* <.01), based on the intraclass correlation coefficient. Test-retest reliability using the Bland-Altman method was fairly good.

**Conclusions:**

This is the first study providing evidence that the Eyes test is reliable and stable over a 1-year period, in a non-clinical sample of adults.

## Background

Psychology researchers have developed reliable instruments for evaluating social cognition and emotional and social processing in both the laboratory and the clinic [1]. Social cognitive studies examine how people process information in the social environment, particularly perceiving, interpreting, and responding to the mental states (intentions, feelings, perception, and beliefs), dispositions, and behaviors of others [2-5]. These processes are tightly linked to processes referred to as emotion recognition and ‘theory of mind’, that allow individuals to imagine the mental state of others [6] to both predict their behavior and respond appropriately. Numerous studies have shown that deficits in emotion recognition and theory of mind compromise social interaction and are related to conditions such as schizophrenia [7,8], autism [9-11], eating disorders [12-14], bipolar disorder [15,16], social anxiety [17], and borderline personality disorder [18].

Different instruments have been developed to assess deficits in social cognition in adults. Instruments designed to assess emotion recognition require the individual to identify emotions and their intensity on the basis of different stimuli, such as facial expressions in the ‘facial emotion identification task’ [19], spoken phrases in the ‘Reading the Mind in the Voice’ test [20] or computer-generated, distorted facial pictures (morphing) [21]. Instruments to assess theory of mind, in contrast, often require individuals to read short stories and answer questions about them [22]. These instruments are intended to assess theory of mind in individuals with autism or Asperger Syndrome, but may also be applicable to other conditions [23].

To provide more detailed information about theory of mind dysfunction, Baron-Cohen *et al*. developed the ‘Reading the Mind in the Eyes’ test, an advanced test of theory of mind [23]. The first version consisted of 25 photographs of actors and actresses showing the facial region around the eyes. The participant is asked to choose which of two words best describes what the person in the photograph is thinking or feeling. These words refer to both basic mental states (for example, ‘happy’) and complex mental states (for example, ‘arrogant’) [23]. In this way, the Eyes test aimed to evaluate social cognition in adults by assessing their ability to recognize the mental state of others using just the expressions around the eyes, which are key in determining mental states [24].

The original ‘Reading the Mind in the Eyes’ test had some limitations because the number of items and the binomial response format did not sufficiently differentiate individuals receiving higher scores. Thus a revised ‘Reading the Mind in the Eyes’ test was created, in which the number of items was increased to 36 and the number of possible responses (single-word descriptors of possible mental states) was increased to 4, reducing the maximum correct guess rate to 25% [1]. The possible mental state descriptors refer mostly to complex mental states. This advanced test was designed to have sufficient analytical complexity to be appropriate for adults with and without psychopathology, brain damage or dementia, to assess factors that might contribute to social difficulties. In this way, the test is intended to allow assessment of social cognition in an adult population with average intelligence.

Although conceived as an advanced theory of mind test [1], the Eyes test is also used to assess emotion recognition. Completing the instrument requires not only the ability to recognize emotional expressions but also the ability to determine the complex cognitive mental state of an individual based on a partial facial expression. Together, these abilities presuppose that the individual possesses a mental state lexicon and knows the meaning of mental state terms [1].

Studies of social cognition impairments in clinical populations show that typical individuals score significantly higher on the Eyes test than do individuals with schizophrenia [7,8], autism [9,10], eating disorders [12,13,25], and social anxiety [17] (for a review, see [26]). These studies indicate that the Eyes test is reliable for assessing social cognition in adults. The Eyes test has also proven useful for assessing social intelligence and its subtle impairment in different cultures, as shown in studies using translations of the Eyes test into Turkish, Hungarian, Japanese, French, German, and Argentinian Spanish [7,27-31].

Most studies with the Eyes test have not reported information on test-retest reliability [26]. This is essential because the Eyes test, like tests explicitly designed to test emotion recognition [32], has psychometric properties that prevent straightforward calculation of Cronbach’s alpha. Calculating this parameter is complex because researchers are limited to comparing the number of correct responses between individuals. Thus, many studies involving the Eyes test do not include Cronbach’s alpha, making it impossible to draw reliable intergroup comparisons, such as comparisons between clinical and control groups or comparisons between the same group before and after an intervention. Intergroup comparisons are also important for cross-cultural studies, which aim to test if cultures differ more in how they identify complex mental states than simpler mental states [26]. Such studies are important for indicating whether the Eyes test should be adapted specifically for different cultures.

Recent studies have addressed this gap by reporting acceptable test-retest stability for the adult version of the Eyes test [26,33] as well as for the child version [34]. The time intervals for retesting in these studies were relatively short, ranging from 2 weeks to 1 month. In order to provide the first assessment of long-term test-retest reliability of the Eyes test, as well as the first detailed validation of the test in a Spanish population, the present study (1) examined the distribution of responses and scores on a Spanish version of the Eyes test in a nonclinical Spanish population, and (2) assessed the 1-year test-retest reliability.

## Method

### Participants

A total of 358 first-year psychology undergraduates enrolled at the Universidad Nacional de Educación a Distancia (UNED, Spain) took part. The sample comprised 75 men and 283 women, with a mean age at the first testing of 34.23 years (sd, 9.02; range, 18 to 65). This bias toward female participants simply reflects the sex ratio in those who choose to study psychology at the undergraduate level. All participants were volunteers who gave written informed consent and who received personalized reports of results at the end of the study. The study was carried out in accordance with the Declaration of Helsinki. Ethics approval was obtained from the Research Ethics Committee, UNED.

### Procedure

The first testing took place during May and June 2011; the second testing took place during the same months in 2012. During both testing sessions, the survey was administered using a computer program that recorded identification data for each participant, displayed test items and saved the responses.

### Measures

The revised Eyes test [1] was used to generate a Spanish version of the Eyes test. Two translators, both with PhDs in psychology and experts in cognition and emotion, created a Spanish version of the instrument, which was then back-translated into English by two independent translators. In this version, as in the English-language original, participants were shown 36 photographs of eye regions of individuals and asked, for each photograph, to choose one of four possible words to describe the mental state of the person shown. One point was assigned for each correct response, so scores could range from 0 to 36. This Spanish version is available from the authors on request.

### Statistical analysis

Data were analyzed using the Statistical Package for the Social Sciences (SPSS), version 19.0. (Armonk, NY: IBM Corp). The Bland-Altman plot to compare test and retest results was calculated using the MedCalc program, version 12.3 (MedCalc™, Mariakerke, Belgium, http://www.medcalc.be). All tests were two-tailed and were conducted at the 5% level of statistical significance.

## Results

Table 1 shows the correct answer for each item on the Spanish version of the Eyes test, and the percentages of participants that selected each answer on the test and retest.

**Table 1:** **Spanish version of the** ‘**Reading the Mind in the Eyes**’ **test**

***Item***	***Answer A***		***Answer B***		***Answer C***		***Answer D***	
	***Test***	***Retest***	***Test***	***Retest***	***Test***	***Retest***	***Test***	***Retest***
1	**66**.**9**	**57**.**1**	17.8	21.4	12.5	17.3	2.8	3.6
2	13.6	10.9	**63**.**8**	**62**.**1**	2.2	2.8	20.3	24.0
3	1.1	1.4	4.5	5.6	**75**.**2**	**71**.**0**	19.2	21.4
4	0.6	2.2	**81**.**1**	**83**.**8**	0.8	0.6	17.0	12.0
5	2.5	2.8	4.7	5.0	**92**.**5**	**91**.**6**	0.3	0.3
6	1.7	2.5	**75**.**2**	**79**.**9**	18.9	14.8	4.2	2.5
7	6.1	5.3	21.7	16.2	**64**.**6**	**71**.**3**	7.2	7.0
8	**88**.**0**	**88**.**3**	4.2	7.2	5.0	3.3	2.8	0.8
9	4.5	6.7	11.4	10.6	2.2	3.3	**81**.**9**	**78**.**8**
10	**71**.**0**	**73**.**0**	21.4	20.9	5.6	3.6	1.7	1.9
11	4.2	3.3	3.9	5.8	**74**.**1**	**72**.**1**	17.8	18.1
12	12.5	12.8	2.2	3.6	**80**.**8**	**79**.**7**	4.5	3.3
13	4.5	4.2	**80**.**8**	**81**.**6**	1.7	1.7	13.1	12.0
14	6.7	8.9	4.2	3.3	0.3	0.3	**88**.**9**	**86**.**9**
15	**86**.**9**	**88**.**6**	9.2	7.5	1.1	1.7	2.5	1.7
16	0.6	1.7	**85**.**8**	**83**.**6**	1.4	2.2	12.0	12.3
17	**54**.**3**	**57**.**1**	27.3	27.9	1.7	1.1	16.7	13.4
18	**96**.**4**	**94**.**7**	1.7	3.6	0.3	0.6	1.7	0.8
19	12.8	9.5	36.2	34.4	12.0	11.1	**39**.**0**	**40**.**4**
20	5.8	4.7	**89**.**4**	**89**.**7**	4.7	5.3	0.0	0.0
21	9.5	8.1	**75**.**2**	**78**.**0**	13.9	13.4	1.1	0.3
22	**70**.**8**	**74**.**1**	0.8	0.6	11.7	9.5	16.4	15.6
23	4.5	3.6	5.8	6.4	**65**.**5**	**69**.**4**	24.2	20.3
24	**73**.**5**	**69**.**1**	8.4	12.0	1.9	2.5	16.2	15.9
25	2.8	3.9	18.7	18.9	8.1	7.2	**70**.**5**	**69**.**4**
26	6.4	5.0	6.7	7.5	**75**.**2**	**76**.**9**	11.4	10.3
27	0.3	0.3	**64**.**1**	**65**.**2**	24.5	20.9	11.1	13.4
28	**83**.**6**	**82**.**5**	1.1	0.8	3.3	5.6	11.4	10.6
29	9.2	3.6	4.5	4.7	5.3	5.0	**81**.**1**	**86**.**4**
30	3.9	3.3	**88**.**6**	**86**.**4**	4.7	7.5	2.8	2.5
31	10.9	10.69	**57**.**1**	**55**.**2**	7.5	8.1	24.2	25.6
32	**78**.**0**	**74**.**4**	4.5	4.7	5.8	9.2	11.4	11.4
33	9.5	10.0	25.1	21.7	4.2	5.3	**61**.**3**	**62**.**4**
34	9.2	12.5	13.6	14.2	**72**.**1**	**68**.**8**	4.5	4.2
35	13.9	11.7	**77**.**7**	**77**.**7**	5.0	7.2	3.1	3.1
36	0.8	0.8	1.4	1.1	**87**.**5**	**89**.**4**	10.0	8.4

Distribution of responses in percentages for test and retest assessment (n = 358).

‘Correct’ responses according to original study are in bold.

Nearly all items on the test were answered correctly by more than 50% of participants. The only exception was item 19, which was answered correctly by only 39.0% of respondents during the test and by only 40.4% during the retest. The next most frequently selected answer B was chosen by 36.2% and 34.4% of respondents during testing and retesting, respectively. The mean percentage of items correctly answered was 75.51% on the test and 75.46% on the retest. There were no significant differences in the percentages of respondents choosing the correct answer across all items during testing and retesting (t = .093, *P* <.926). In fact, for all items, the correct answer was chosen far more often than the next most frequently selected option.

**Figure 1:**
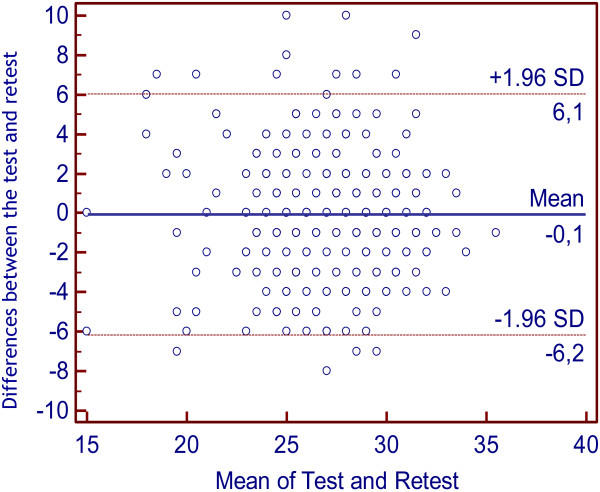
**Bland**-**Altman plot of the eyes test**-**retest assessment (n) = 358.**

Converting the mean percentages above to the 0 to 36 scale of the Eyes test gave mean point scores of 27.18 (sd = 3.59) on the test and 27.24 (sd = 3.67) on the retest (t = .36, *P* <.722). Test-retest stability was assessed using the intraclass correlation coefficient (ICC), which was .63 for the total score (P <.01). Table 2 shows test-retest correlations for each of the items. Correlations for all items except item 18 were positive and significant. Although results for item 18 showed 330 of 358 possible coincidences, no significant linear correlation was found. This result does not mean that test and retest results for item 18 were independent, but rather that they were not linearly related. The Bland-Altman plot [35] was used to examine test-retest concordance. This graphical approach allows for the examination of the agreement between repeated measurements by plotting the differences between test and retest scores against the mean value of the test and retest scores for each participant. Confidence intervals for the mean difference are calculated to determine if the mean difference deviates significantly from zero (Figure 1).

**Table 2:** **Spearman**’**s Rho correlations between test and retest for each item for right and wrong answers** (**n** = **358**)

**Item**	***Spearman***’***s Rho correlation***
1	.391^**^
2	.297^**^
3	.278^**^
4	.197^**^
5	.226^**^
6	.204^**^
7	.144^**^
8	.151^**^
9	.390^**^
10	.323^**^
11	.248^**^
12	.183^**^
13	.253^**^
14	.276^**^
15	.159^**^
16	.274^**^
17	.252^**^
18	.095
19	.201^**^
20	.110^*^
21	.192^**^
22	.278^**^
23	.189^**^
24	.277^**^
25	.289^**^
26	.129^*^
27	.179^**^
28	.197^**^
29	.207^**^
30	.190^**^
31	.124^*^
32	.306^**^
33	.231^**^
34	.162^**^
35	.184^**^
36	.115^*^

*P <.05, **P <.01.

The mean difference between test and retest responses across all participants was −0.06 (*SD* = 3.12), indicating no significant change in results between testing and retesting one year later. The 95% confidence interval (CI) for the mean difference was −6.17 to 6.05; thus, the CI included 0. Most results fell within the 95% CI, and those that did not failed to show any tendency, suggesting that they reflected chance variation. Estimated measurement error based on within-subject standard deviation was 3.63, and the coefficient of repeatability was 6.24.

## Discussion

The primary purpose of the current study was to examine the long-term reliability of the Spanish translation of the Eyes test in Spain. To our knowledge, this is the first study providing evidence that the Eyes test is reliable and stable over a 1-year period in a nonclinical population sample. To determine the reliability of the Spanish version of the Eye test, we analyzed the distribution of responses for each item during testing and retesting one year later. The results indicate that not all items are equally difficult, which should increase the discriminant ability of the test. The distribution of difficulty across all items of the test was approximately normal and greater than 50% for the correct response. Despite the fact that less than 50% of the respondents correctly answered item 19, the majority did in fact choose the correct answer. In the Italian version of Eyes test similar percentages were obtained [26]. Further research should be conducted to determine if the item should be eliminated due to ambiguity or retained because it is difficult, and therefore useful in testing emotional discrimination.

Test-retest reliability using the ICC indicated a significant correlation between the total scores on the test and retest, demonstrating that results were stable over time. They also indicate that no learning occurred in the study population [34]. Item-by-item correlation analysis between test and retest showed that responses to all items except item 18 were stable over time. This finding implies that emotion recognition judgments, both correct and incorrect, persist over time. The relatively long interval of 1 year between test and retest further suggests that such persistence is not due to chance but to the existence of stable cognitive dispositions in recognizing complex emotions [1].

We used the graphical method of Bland-Altman to assess test-retest concordance on our Spanish translation of the Eyes test. This approach allowed us to analyze the position of test-retest differences relative to the test-retest mean. This analysis showed that most responses on all items were concordant with one another; mean differences were 0, and most differences fell within the 95% CI. The differences were homogeneous and appeared to be distributed randomly across all items of the test, with no evidence of a systematic bias or tendency. The small differences and their homogeneity lead us to conclude that the Eyes test is reliable and stable for up to 1 year, not only with respect to total scores but also to the distributions of answers for each item. These results may help guide the identification of items that discriminate between clinical and nonclinical populations in further studies.

This study is not without limitations. First, the proportion of women in our test population was much higher than that of men, raising the possibility of gender bias. Second, this study examined test-retest reliability over a relatively long period of 1 year. Future studies should also investigate the stability of the Spanish version over shorter time periods, since stability is expected to be greater over shorter periods [26].

Several studies using Eyes test have analyzed gender and age differences without conclusive results [26]. Our study did not address these issues. Future studies should investigate these differences and explore the mechanisms by which gender and age influence the development of theory of mind and emotional recognition. Additionally, it would be interesting to examine how other objective measures of emotion recognition, empathy, and emotional intelligence are related with Eyes test.

Numerous international studies using the Eyes test have shown group differences in emotion recognition and theory of mind between individuals diagnosed with schizophrenia [7,8] or autism [9-11] and typical control groups. The Spanish version of the Eyes test will help in the diagnosis and effective implementation of intervention programs for individuals with impairment in social cognition in Spanish-speaking countries. This test will allow the comparison of an individual’s score with the normative scores of Spanish samples and will enable researchers and clinicians to describe with accuracy any change of their scores before and after intervention programs.

## Conclusions

In conclusion, the results from the current study suggest that the Eyes test is a reliable measure of theory of mind and recognition of complex emotions in adults, and that it is stable over a 1-year period in a nonclinical population. This Spanish version of the Eyes test will be useful in future research into social cognition in laboratory and clinical contexts, including cross-cultural and clinical investigations into autism and related neurodevelopmental conditions, in Spain and in other Spanish-speaking countries.

## Abbreviations

CI: Confidence interval; ICC: Intraclass correlation coefficient; sd: Standard deviation.

## Competing interests

The authors declare they have no competing interests.

## Authors’ contributions

EGFA conceived of the study, participated in the data collection, analyzed the data and led preparation of the manuscript. RC and PFB conceived of the study and wrote the first draft of the manuscript. SBC contributed to writing the manuscript. All authors contributed to the interpretation of data, helped to draft and revise the manuscript and have read and approved the final manuscript.
